# Virus Diversity, Abundance, and Evolution in Three Different Bat Colonies in Switzerland

**DOI:** 10.3390/v14091911

**Published:** 2022-08-29

**Authors:** Myriam Anja Wiederkehr, Weihong Qi, Katja Schoenbaechler, Cornel Fraefel, Jakub Kubacki

**Affiliations:** 1 Institute of Virology, University of Zurich, 8057 Zurich, Switzerland; 2 Functional Genomics Center Zurich, Swiss Federal Institute of Technology (ETH Zurich), University of Zurich, 8057 Zurich, Switzerland; 3 Bat Foundation Switzerland, 8044 Zurich, Switzerland

**Keywords:** bats, viral metagenomics, Switzerland, virus, diversity, mutations, natural environment, coronavirus, rotavirus, reservoir host, virus evolution

## Abstract

Bats are increasingly recognized as reservoirs for many different viruses that threaten public health, such as Hendravirus, Ebolavirus, Nipahvirus, and SARS- and MERS-coronavirus. To assess spillover risk, viromes of bats from different parts of the world have been investigated in the past. As opposed to most of these prior studies, which determined the bat virome at a single time point, the current work was performed to monitor changes over time. Specifically, fecal samples of three endemic Swiss bat colonies consisting of three different bat species were collected over three years and analyzed using next-generation sequencing. Furthermore, single nucleotide variants of selected DNA and RNA viruses were analyzed to investigate virus genome evolution. In total, sequences of 22 different virus families were found, of which 13 are known to infect vertebrates. Most interestingly, in a *Vespertilio murinus* colony, sequences from a MERS-related beta-coronavirus were consistently detected over three consecutive years, which allowed us to investigate viral genome evolution in a natural reservoir host.

## 1. Introduction

It has been estimated that up to 75% of emerging and reemerging infectious diseases originate from wildlife species [1,2]. The increase of zoonotic spillover is likely due to human population growth, high exposure of humans to wildlife and products of animal origin, intensive livestock farming, climate change, habitat destruction, wildlife markets, and bushmeat consumption [3,4,5,6,7]. The inter- and intraspecies transmission of infectious diseases is based strongly on interactions between hosts, pathogens, and the environment [5].

Bats are considered to play a major role in disease emergence and as reservoir hosts of viruses that pose a risk to public health [8,9,10,11]. Most of the viruses found in bat feces originate from insects, plants, and fungi, representing the dietary habits of the animals [12]. However, many viruses of vertebrates are also found in bats, including emerging viruses such as Hendravirus, Ebolavirus, Nipahvirus, and SARS- and MERS-coronavirus (CoV) [12,13,14,15,16,17,18]. The origin of SARS-CoV-2 has not yet been clarified. However, in several different animal species, i.e., Himalayan palm civets, Asian palm civets, and bats of the species *Rhinolophus* and *Aselliscus,* viruses closely related to SARS-CoV-2 have been detected [19]. Although a beta-coronavirus named RaTG13 isolated from a bat of the species *Rhinolophus affinis* in Yunnan province in China seems to be the virus most closely related to SARS-CoV-2, with 96.2% nucleotide identity [20], this virus is not assumed to be the direct progenitor of SARS-CoV-2 [20,21]. SARS-CoV-2 is suspected to have a history of recombination and to share a common ancestor with three other bat viruses called PRC31, RpYN06, and RmYN02, which are more similar than RaTG13 in most of the genome [22,23].

Several factors can facilitate the spillover of viruses from bats to other animals and/or humans. Intrinsic factors such as body condition, reproduction status, age, and sex can play a role, as well as extrinsic factors leading to habitat change, such as climate change, loss of habitat, lack of food, and anthropogenic factors [2,24,25]. Furthermore, parturition and breeding can lead to peaks of viral shedding caused by (i) the accumulation of many bats in the maternity roosts and (ii) the establishment of a susceptible subpopulation of newborn bats without immunity [26].

Approximately 51 different bat species have been identified in Europe, of which 30 species from 4 different families, i.e., *Vespertilionidae, Rhinolophidae, Miniopteridae*, and *Molossidae*, are endemic in Switzerland [27,28,29,30,31]. Five endemic Swiss bat species, i.e., *Nyctalus noctula, Nyctalus leisleri, Nyctalus lasiopterus, Vespertilio murinus*, and *Pipistrellus nathusii*, are migratory and are known to fly long distances [27]. In this study we focused particularly on the two non-migratory species *Rhinolophus hipposideros* and *Myotis myotis*, and on one migratory bat species, *Vespertilio murinus* [27]. *Rhinolophus hipposideros* flies approx. 2.5 km for hunting and 20 km for hibernation in winter roosts, while *Myotis myotis* covers approx. 20 km for hunting and up to 100 km for hibernation in winter roosts [27]. *Vespertilio murinus* migrates over 800 km from summer to winter [27].

Next-generation sequencing (NGS) has been used to investigate the virus diversity of various bat species in many different countries worldwide [31,32,33,34,35,36,37,38,39]. However, most of the studies sampled at a single time point, and the few studies that sampled at different time points did not assess the change in the virome over time [40], sampled at very short intervals of three days [12] or two weeks [41], or did not analyze the genomes at the single-nucleotide level [26,42,43,44].

The only metagenomic study of bats previously performed in Switzerland reported the virome of 18 different bat species, providing a general overview of the viruses at different locations and sample types [39]. For the present study, we re-sampled selected colonies from that pilot study over two additional years to investigate the changes in the virome composition and—for selected viruses, alpha- and beta-CoVs in particular—the evolution of the genome sequence in the natural reservoir species. Virus evolution in the natural host likely differs fundamentally between different species and from that observed upon passaging in cell culture at standardized conditions, as many different internal and external factors influence both the host and the virus, including stress, environmental conditions, and the presence of other infectious agents.

## 2. Materials and Methods

### 2.1. Sample Type

Based on a previous virome study of Swiss bats [39], three colonies of different bat species were selected for further investigation in the present study. The selected colonies were located in the cantons of Aargau (AG), Grisons (GR), and Lucerne (LU). The geographic coordinates of bat colonies can be provided by the Swiss Bat Conservation Foundation upon formal request. The samples were collected by the local caretakers in May 2019, 2020, and 2021 from the AG and LU colonies, and in the years 2019 and 2021 from the GR colony. The AG colony consisted of approx. 150 to 200 *Vespertilio murinus* bats, the GR colony of approx. 30 *Rhinolphus hipposideros* bats, and the LU colony of approx. 520 *Myotis myotis* bats.

### 2.2. Sample Preparation and Collection

Fecal samples (fully filled 50 mL tubes) from bat colonies were collected from various places on the floor during routine inspection in May by authorized persons when the animals were present; 15 mL conical tubes fully filled with fecal samples were mixed 1:1 with phosphate buffered saline (PBS), vortexed vigorously, and homogenized overnight (Hu-laMixerTM Sample Mixer, Life Technologies, Carlsbad, CA, USA) at 4 °C. The tubes were then centrifuged for 10 min at 5903× *g* (Hereus Multifuge 3 S-R, Thermo Fisher, Waltham, Massachusetts, USA), and 1 mL of the supernatant was transferred to a 2 mL tube and processed following an in-house protocol as described previously [39,45]. Briefly, the supernatant was enriched for viral particles by centrifugation, filtration, and nuclease treatment, which was followed by nucleic acid extraction, reverse transcription, second-strand synthesis, and unspecific DNA amplification.

DNA was fragmented to 500 bp by sonication (E220 Ultrasonicator, Covaris, USA). Sequencing libraries were prepared using a NEBNext Ultra II DNA Library Prep Kit for Illumina (New England Biolabs, Ipswich, MA, USA), cleaned with AMPure XP beads (Beckman Coulter, Brea, CA, USA), and barcoded with NEBNext Multiplex Oligos (96 Unique Dual Index Primer Pairs; New England Biolabs, Ipswich, MA, USA). The molarity and size distribution of the libraries was determined on an Agilent 2200 TapeStation using a D1000 HS ScreenTape (Agilent Technologies, Santa Clara, CA, USA). Sequencing was carried out at the Functional Genomics Center Zurich (FGCZ) using an Illumina NovaSeq 6000 Benchtop sequencer (Illumina, San Diego, CA, USA) in paired-end NGS runs of 2 × 150 nucleotide read length. As sequencing control, PhiX Control v3 Library (Illumina, San Diego, CA, USA) was used.

### 2.3. Virome Analysis

The generated raw sequences were analyzed by de novo assembly and reference-guided in-house assembly pipelines as described previously [39,46]. Briefly, the Illumina sequencing adapters (default), low-quality sequencing ends, and SISPA primers were trimmed using Trimmomatic (v0.39) and cutadapt (v3.2) [47]. Subsequently, quality-proofed reads were assembled using metaspades (v3.12.0) and compared to the NCBI non-redundant database using blastn (v2.10.1+) [48]. Alignment summary statistics per contig were collected by running samtools (v1.11) idxstats. Finally, trimmed reads were aligned (cut-off: 10 reads over the genome) in a metagenomic pipeline of the SeqMan NGen v.17 software (DNAStar, Lasergene, Madison, WI, USA) to an in-house database containing over 60,000 full-length virus genomes downloaded from the NCBI database and bat-associated viruses from the DBatVir database [49].

#### 2.3.1. Comparison of Viruses and Genes

Reads were cleaned from adapters and quality-trimmed with fastp (v0.20.1, [50]). Reads matching ribosomal sequences were removed with sortmerna (v4.3.4, [51]) using the eukaryotic 18S and 28S reference sequences from SILVA [52]. Reads matching bat genomes from *Myotis myotis* (NCBI ID GCF_014108235.1) or *Rhinolophus ferrumequinum* (NCBI ID GCF_004115265.1) with minimap2 (v2.24-r1122, [53]) were removed as well. Surviving reads were re-paired with the repair.sh script from BBTools (https://sourceforge.net/projects/bbmap, accessed on 10 July 2022). Only paired reads were kept and aligned to a collection of viral reference sequences (downloaded from NCBI on February 2022; see Table S1) using bowtie2 with the option --very-sensitive-local (v2.3.5.1, [54]). The reference sequences were specific for each colony and were based on the results from the virome analysis above. Single nucleotide variants (SNVs) were called with freebayes (v1.3.2-40-gcce27fc, [55]) with the options -p 1 --min-coverage 10 --skip-coverage 100000 --min-alternate-count 2 --use-best-n-alleles 2 --min-alternate-fraction 0.1 --pooled-continuous --min-mapping-quality 1. SNV calls were filtered for a minimal quality of 20 with the script vcffilter distributed with freebayes. Number of reads and percent genome coverage were extracted with samtools (v1.10, [56]). The SNVs were filtered to include only the two best-supported variants per site. Importantly, these were not necessarily the reference SNVs from the reference assembly. Hence, we did not classify any of them as reference SNVs, but only compared the change in SNV frequencies over time.

#### 2.3.2. Test for Shifts in SNV Frequency

To test whether SNVs changed in frequency from one year to the later years, we compared the allelic depths with Fisher’s exact test. We did this for 2019 vs. 2020, 2020 vs. 2021, and 2019 vs. 2021. The *p*-values from all comparisons were then adjusted to reflect false discovery rates (FDRs), and sites with an FDR < 0.01 were marked as significant. We classified whether the SNVs had an effect on the coding sequence by comparing the protein sequences containing the two best-supported SNVs with a custom script. We also identified potentially novel SNVs by checking whether variants were already present in the earlier year (s).

### 2.4. Phylogenetic Analysis

The NSP12 genes (encoding RNA-dependent RNA polymerase (*RdRp*)) of the alpha- and beta-CoVs identified in this study were aligned using MUSCLE in MEGA X [57]. Phylogenetic trees were constructed in Mega X using the Maximum Likelihood algorithm based on the Tamura–Nei model with 1000 bootstrap values and a cut-off of 70%. For both viruses, full NSP12 reference genes were downloaded from the NCBI sequence database based on BLAST analysis.

### 2.5. PCR

The presence of a Middle East respiratory syndrome-related coronavirus (MERS-related CoV) detected by NGS each year in the AG colony was confirmed by RT-PCR targeting the entire gene encoding the spike protein. First, RNA was extracted using the QIAamp Viral RNA Mini Kit (Qiagen, Hombrechtikon, Switzerland) according to the manufacturer’s manual with a sample volume of 140 µL and the following modifications: RNA carrier was omitted, and for the elution step, 100 µL RNase free water was used instead of 60 µL AVE buffer. Then, the cDNA was synthesized using the SuperScript® IV First-Strand Synthesis System kit (Thermo Fisher, Waltham, MA, USA) as described by the manufacturer using 11 µL of RNA and random hexamers (Thermo Fisher, Waltham, MA, USA). To amplify the genomic region of the spike protein, five overlapping pairs of specific primers were designed using the Primer-BLAST tool (https://www.ncbi.nlm.nih.gov/tools/primer-blast/, accessed on 10 July 2022) based on the sequences obtained from NGS. Additional primer pairs were designed to fill gaps based on the Sanger sequences (Table S2). PCR was performed using a Phusion Hot Start II High Fidelity DNA Polymerase kit (Thermo Scientific, Waltham, MA, USA) in a three-step protocol (as described in the manual provided by the manufacturer) with 45 cycles and 1.5 µL of cDNA in a final reaction volume of 25 µL. The PCR products were visualized on 1.5% agarose gel with GelRed® (GelRed® Nucleic Acid Gel Stain, Biotium, Fremont, CA, USA) staining. PCR products with the expected size were purified by a QIAquick PCR Purification Kit (Qiagen, Hombrechtikon, Switzerland) following the instructions of the manufacturer with an elution volume of 30 µL. Finally, the DNA concentration was measured on the NanoDropTM OneC (Thermo Fisher Scientific, Waltham, MA, USA), and sent to Microsynth (Balgach, Switzerland) for Sanger sequencing.

### 2.6. Data Availability

The nucleotide sequences of the coronaviruses detected in this study were registered at GenBank under accession numbers ON325307–ON325310. All raw sequencing data were uploaded to the Sequence Read Archive (SRA) under accession number PRJNA835632.

## 3. Results

The results present the unbiased virome of three bat colonies and the evolution of selected virus genomes over three years.

### 3.1. Virome Analysis

**Figure 1:**
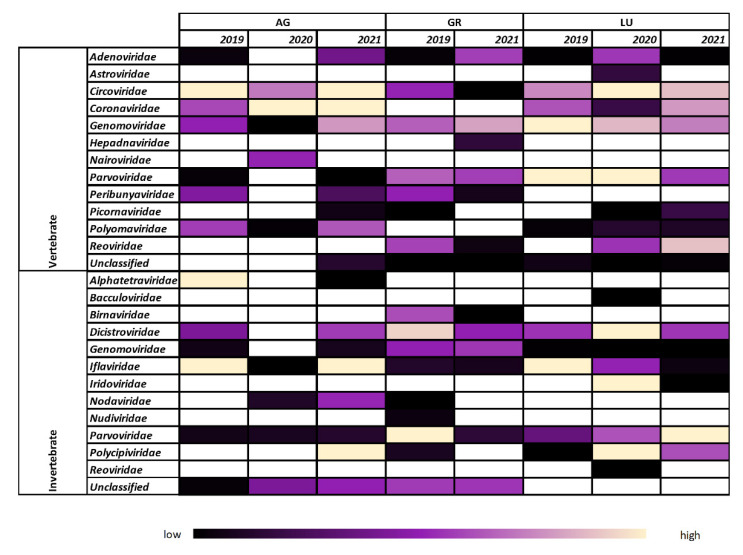
Overview of virus families from vertebrates and invertebrates detected in the Aargau (AG), Grisons (GR), and Lucerne (LU) colonies. The heatmap represents the relative number of viral reads from black (15th percentile of the lowest number) to purple (50th percentile) to light yellow (85th percentiles of the highest number). White = not detected.

**Figure 2:**
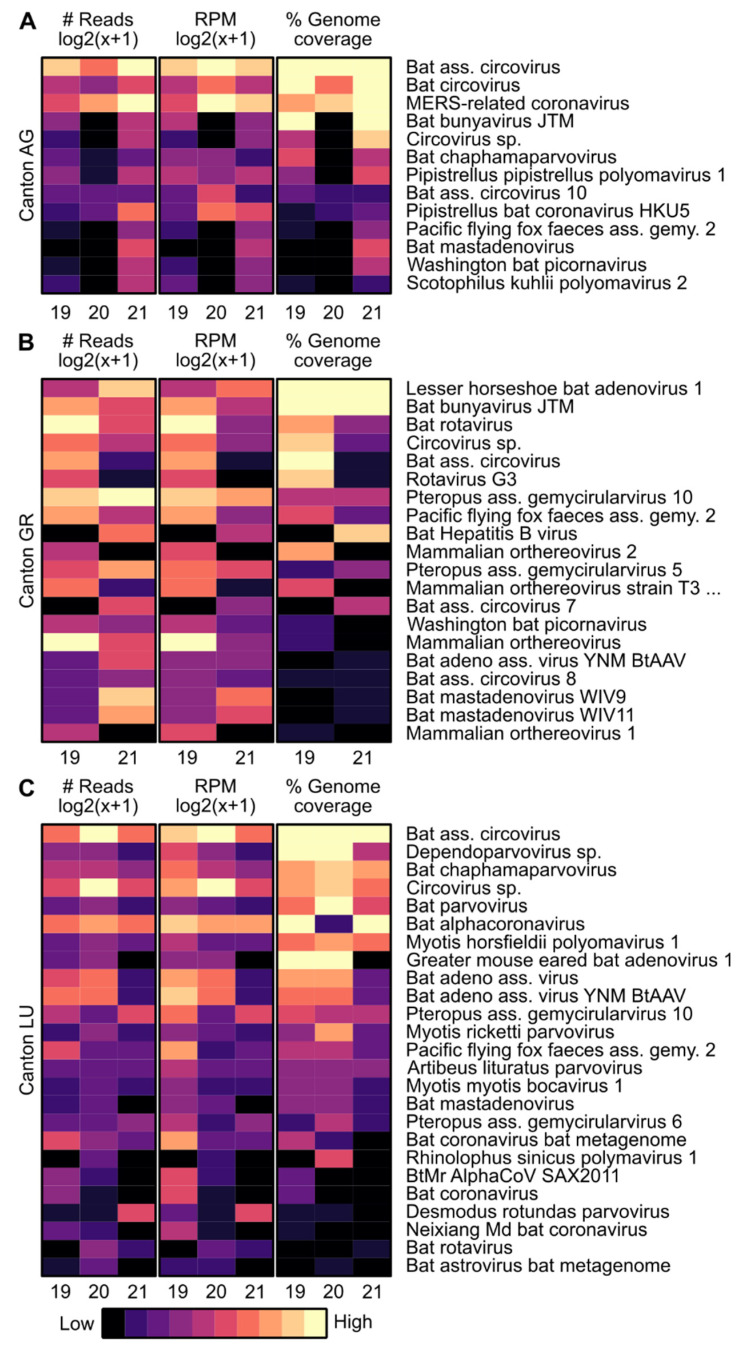
Heatmaps of specific genomes of viruses from vertebrates detected in (**A**) AG, (**B**) GR, and (**C**) LU colonies aligned to a collection of reference sequences. The first column shows the number of reads on a logarithmic scale, the second reads per million (RPM), and the third the percent genome coverage. The read numbers are represented in a gradient from black (low numbers) to light yellow (high numbers). For percent genome coverage, black indicates 0% and light-yellow 100%.

In total, 1.69 × 10^8^ raw sequencing reads were generated with an average of 2.17 × 10^7^ reads per sample (range from 1.27 × 10^7^ to 3.27 × 10^7^ reads per sample) and analyzed using a reference-based assembly. The raw sequencing reads also included sequences of bacteriophages and plant viruses, which were excluded from further analysis. From the total number of reads, 2.76 × 10^6^ (1.63%) were classified as viral, of which 1.57 × 10^6^ (57%) were determined as viruses from vertebrates and 1.19 × 10^6^ (43%) as viruses from invertebrates. Figure 1 shows a general overview of the different virus families from vertebrates and invertebrates found in the different colonies and years. Figure 2 and Supplementary Figures S1–S3 represent heatmaps of specific viruses of each colony aligned to a collection of viral reference sequences. The reads classified as viral were assembled to 22 different virus families, i.e., 9 from vertebrates, 9 from invertebrates, and 4 families with members infecting vertebrates or invertebrates.

The virus sequences identified at least at one sampling time point in the AG colony belonging to 15 different virus families (Figure 1). Of these, seven families are known to infect vertebrates, including *Adenoviridae, Cirvoviridae, Coronaviridae, Nairoviridae, Peribunyaviridae, Picornaviridae*, and *Polyomaviridae*, and five families are known to infect invertebrates, including *Alphatetraviridae*, *Dicistroviridae*, *Iflaviridae*, *Nodaviridae*, and *Polycipiviridae*. Three of the virus families found include members that can infect vertebrates or invertebrates, including *Genomoviridae, Parvoviridae*, and unclassified viruses. Five families of DNA viruses were identified, i.e., *Adenoviridae, Circoviridae, Genomoviridae, Parvoviridae*, and *Polyomaviridae,* along with nine families of RNA viruses, i.e., *Coronaviridae, Nairoviridae, Peribunyaviridae, Picornaviridae, Alphatetraviridae, Dicistroviridae, Iflaviridae, Nodaviridae*, and *Polycipiviridae*. The unclassified viruses included both RNA and DNA viruses. Sequence reads from seven different virus families, i.e., *Circoviridae, Coronaviridae, Genomoviridae*, *Polyomaviridae, Iflaviridae, Parvoviridae*, and unclassified viruses, were found at all three time points. Seven others, including *Adenoviridae, Genomoviridae*, *Peribunyaviridae, Parvoviridae*, *Alphatetraviridae, Dicistroviridae*, and *Nodaviridae,* were only found at two time points. The remaining four, including *Nairoviridae, Picornaviridae*, *Polycipiviridae*, and unclassified viruses from vertebrates, were detected at just a single time point. Among the sequences from viruses infecting vertebrates, reads from *Coronaviridae* and *Circoviridae* were the most abundant. Specifically, sequences from a MERS-related CoV that was detected in this colony in 2019 [39] were also detected in high numbers in 2020 and 2021. Reads from bat-associated circovirus (aligned to MT815982) were also found at all three time points and at high numbers (Figure 2A). Among the reads from viruses infecting invertebrates, *Iflaviridae, Alphatetraviridae*, and *Polycipiviridae* were the most abundant, although the read numbers varied greatly between the different sampling time points (Figure 1 and Figure S1).

In the GR colony, viral reads belonging to 15 different families were identified for at least at one sampling time point (Figure 1). Although this number is identical to that of the AG colony, the specific virus families only partial overlap. Of the 15 families, 6 are known to infect vertebrates, including *Adenoviridae*, *Circoviridae*, *Hepadnaviridae*, *Peribunyaviridae*, *Picornaviridae, and Reoviridae*, and 6 families are known to infect invertebrates, including *Birnaviridae*, *Dicistroviridae*, *Iflaviridae*, *Nodaviridae*, *Nudiviridae*, and *Polycipiviridae*. Three of the virus families found include members that can infect vertebrates or invertebrates, including *Genomoviridae*, *Parvoviridae*, and unclassified viruses. Six families of DNA viruses were identified, i.e., *Adenoviridae, Circoviridae, Genomoviridae, Hepadnaviridae, Parvoviridae, and Nudiviridae*, and eight families of RNA viruses, i.e., *Peribunyaviridae, Picornaviridae, Reoviridae, Birnaviridae, Dicistroviridae, Iflaviridae, Nodaviridae*, and *Polycipiviridae*. The unclassified viruses included both RNA and DNA viruses. Ten virus families, i.e., *Adenoviridae, Circoviridae, Genomoviridae, Parvoviridae, Peribunyaviridae, Reoviridae, Birnaviridae, Dicistroviridae, Iflaviridae*, and unclassified viruses, were detected at both collection time points, whereas five virus families, including *Hepadnaviridae, Picornaviridae, Nodaviridae, Nudiviridae*, and *Polycipiviridae,* were just found at a single time point. Among sequences from viruses infecting vertebrates, reads from the *Genomoviridae* family, specifically Pteropus-associated gemycircularvirus 10 (aligned to NC_038493), were the most abundant (Figure 1 and Figure 2B). Among the reads from viruses infecting invertebrates, the *Parvoviridae* family (mainly aligned to mosquito densovirus, NC_015115) was most abundant (Figure S2).

The number of different virus families identified in the LU colony was 15, as in the AG and GR colonies, and again, the different families only partially overlapped (Figure 1). Of these, seven families are known to infect vertebrates, including *Adenoviridae, Astroviridae, Circoviridae, Coronaviridae, Picornaviridae*, *Polyomaviridae*, and unclassified viruses, and five families are known to infect invertebrates, including *Bacculoviridae, Dicicstroviridae, Iflaviridae, Iridoviridae*, and *Polycipiviridae*. Three of the virus families found include members that can infect vertebrates or invertebrates, including *Genomoviridae, Parvoviridae*, and *Reoviridae*. Seven families of DNA viruses were identified, i.e., *Adenoviridae, Circoviridae, Genomoviridae, Parvoviridae, Polyomaviridae, Bacculoviridae*, and *Iridoviridae*, along with seven families of RNA viruses, i.e., *Astroviridae, Coronaviridae, Picornaviridae, Reoviridae, Dicistroviridae, Iflaviridae*, and *Polycipiviridae*. The unclassified viruses included both RNA and DNA viruses. Sequence reads from ten different virus families, i.e., *Adenoviridae, Circoviridae, Coronaviridae, Genomoviridae, Parvoviridae, Polyomaviridae, Dicistroviridae, Iflaviridae, Polycipiviridae*, and unclassified viruses, were detected at all three time points. Sequence reads from three different virus families, i.e., *Picornaviridae, Iridoviridae*, and *Reoviridae* from vertebrates, were found at two time points. The remaining virus families, *Bacculoviridae, Astroviridae*, and *Reoviridae* from invertebrates, were found at a single time point. Among the sequences from viruses infecting vertebrates, reads from *Genomoviridae*, *Parvoviridae*, and *Circoviridae* were the most abundant. One of the viruses with a constantly high read abundance over the years was a CoV belonging to the genus *alpha-coronavirus* (aligned to MN065811) (Figure 2C). Although the reads per million (RPM) was consistently high, the genome coverage in the year 2020 was poor.

As in the AG colony, bat-associated circovirus (aligned to MT815980) was the most abundant virus from vertebrates and showed the same RPM pattern, with the highest number in 2020 (Figure 2C).

### 3.2. Variability of Selected Genomes and ORFs over Time

We analyzed SNVs and amino acid substitutions in single-sequence reads of selected DNA and RNA viruses of vertebrates relative to reference genomes (Table 1 and Table 2).

#### 3.2.1. Variability of Selected DNA Virus Genomes

In the AG colony, sequences aligned to two different circoviruses (bat circovirus isolate Acheng30, NC_035799, and bat-associated circovirus isolate BatACV/BtVm/Switzerland/2019, MT815982) and one polyomavirus (pipistrellus pipistrellus polyomavirus 1 PyV8-SHZC27, LC426677) were analyzed (Table 1). In the gene for the replication protein (*rep*) of the bat-associated circovirus, only three non-synonymous nucleotide substitution were detected out of a total of 13 substitutions. In the sequences encoding the late T-antigen of the polyomavirus, only a single synonymous nucleotide substitution was observed.

In the GR colony, changes in the sequences aligned to adeno-associated virus 2 (AAV2, AF043303) and starling circovirus (DQ172906) were analyzed (Table 1). In four genes of AAV2, i.e., *rep68*, *78*, *40,* and *52*, two non-synonymous nucleotide substitutions per gene were identified. In the circovirus, only one synonymous nucleotide substitution in the capsid protein (*cap*) was found.

In the LU colony, sequences aligned to five different viruses, i.e., cyclovirus TsCyV-1_JP-NUBS-2014 (NC_027530), circovirus sp. strain UK03/Ukr/2014 (KY302871), dependoparvovirus sp. strain Neo1306140 (MF579866), bat AAV isolate 09YN (MH167452), and murine-associated porcine bocavirus isolate MAPBV/NYC/2014/M074/0626 (MF175076) were analyzed (Table 1). In the *rep* genes of both the cyclovirus and the circovirus, six nucleotide substitutions were detected. All substitutions in the cyclovirus *rep* sequences were non-synonymous, while three of the six substitutions in the circovirus genome were non-synonymous. In dependoparvovirus, four nucleotide substitutions were detected, of which one was non-synonymous. The *cap* gene of bat AAV had two non-synonymous mutations. In bocavirus NP1, VP1, and VP2 genes, none of the seven detected nucleotide substitutions resulted in an amino-acid change.

#### 3.2.2. Variability of Selected RNA Virus Genomes

**Figure 3:**
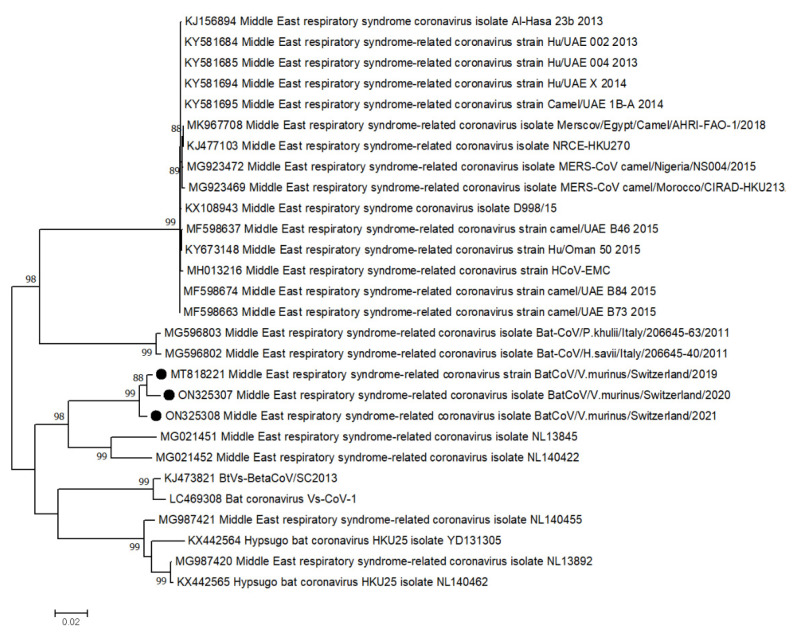
Phylogenetic tree based on nucleotide sequence identity of the NS12 protein (which includes RNA-dependent RNA polymerase (*RdRp*)) of selected beta-coronaviruses. Sequences found in this study are marked by black dots. Phylogenetic analysis was performed using the Maximum likelihood algorithm based on the Tamura–Nei model with the 1000 replications bootstrap method. Only values ≥ 70% are displayed. Evolutionary analyses were conducted in MEGA X.

In the AG colony, sequences aligned to a MERS-related CoV (MG021452) were found at all three time points. According to phylogenetic analyses based on the RNA-dependent RNA polymerase (*RdRp*) gene, this virus sequence clustered with and showed the highest similarity (approx. 87%) to a MERS-related CoV genome from China (MG021452) (Figure 3).

As there was some variation of the genome coverage over the three years—77% in 2019, 86% in 2020, and 91% in 2021—only sequences that were identified in at least two years were analyzed for SNVs. Of the 186 substitutions found in the *ORF1ab* gene, 14 were non-synonymous and 14 were novel (Table 2). A substitution was classified as novel when it was not previously present in the dataset. In the gene encoding the spike (S) protein, 12 substitutions were identified, of which 3 were non-synonymous and 3 were novel. In *ORF4a* and *b*, one non-synonymous substitution was detected in each gene, and in both genes, one substitution was novel. In *ORF5*, one out of four substitutions was non-synonymous and one substitution was novel. In genes encoding the envelope (E) and membrane (M) proteins, all substitutions were synonymous, while for the gene encoding the nucleocapsid (N) protein, 2 out of 10 substitutions detected were non-synonymous and 2 were novel. Finally, for *ORF8b*, five substitutions were identified, of which four were non-synonymous and three were novel (Table 2).

**Figure 4:**
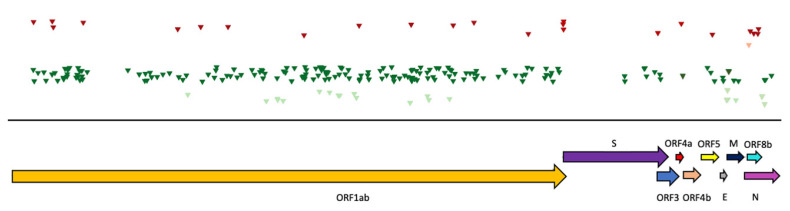
Overview of single nucleotide variants (SNVs) distributed over the genome of MERS-related-CoV from the AG colony. Arrows show the position of the ORFs. Each triangle indicates an SNV; light green, synonymous and novel; dark green, synonymous; light red, non-synonymous and novel; dark red, non-synonymous.

**Figure 5:**
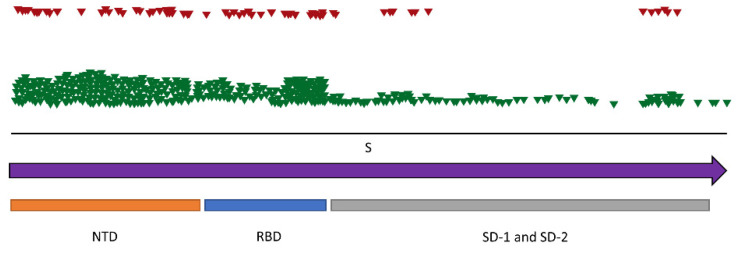
Overview of SNVs distributed over the spike protein locus (arrow) of MERS-related-CoV from the AG colony based on RT-PCR and Sanger sequencing. The domains (N-terminal domain of the S1 subunit (*NTD*); receptor-binding domain of the S1 subunit (*RBD*); subdomains of S1 and S2 subunits (*SD-1 and SD-2*)) were predicted by ClustalW alignment to the homologous regions of available MERS-related CoVs. Each triangle indicates an SNV; green, synonymous; red, non-synonymous.

The substitutions were distributed equally over the genome, except for the locus encoding the spike protein and the *ORF3*, which showed a lower number of substitutions (Figure 4). However, this is likely an artifact because the genomic region was only covered in the NGS data from the 2020 sample. To overcome this issue, we RT-PCR amplified and Sanger sequenced the genomic region encoding the spike protein from samples collected at all three time points. The analysis revealed that of the 429 substitutions, 333 were synonymous and 96 were non-synonymous. The majority of substitutions were found in the N-terminal domain (*NTD*) and the receptor-binding domain (*RBD*), while there were fewer in the S1 and S2 subdomains (*SD-1 and SD-2*) (Figure 5). The non-synonymous substitutions were equally distributed over the regions of *NTD* and *RBD*, whereas the distribution of non-synonymous substitutions in the region of the *SD-1 and SD-2* showed a gap.

The MERS-related CoV contig of 2019 had previously been uploaded to GenBank (MT818221). The contig of 2020, which was covered completely (30’047 nt), and of 2021, which was partial (25’618 nt), are uploaded now, as well, and can be found under accession numbers ON325307 and ON325308, respectively.

In the GR colony, one synonymous nucleotide substitution was identified in each of two bat rotavirus segments encoding *NSP3* (aligned to MN433625) and *VP4* (aligned to MN433620). In the two mammalian orthoreovirus segments sequenced, one substitution was synonymous (*L3*, aligned to KU194660), and the other was non-synonymous and also novel (*S1*, aligned to JQ979272) (Table 2). However, it is important to mention that the coverage for both viruses in the year 2021 was low.

**Figure 6:**
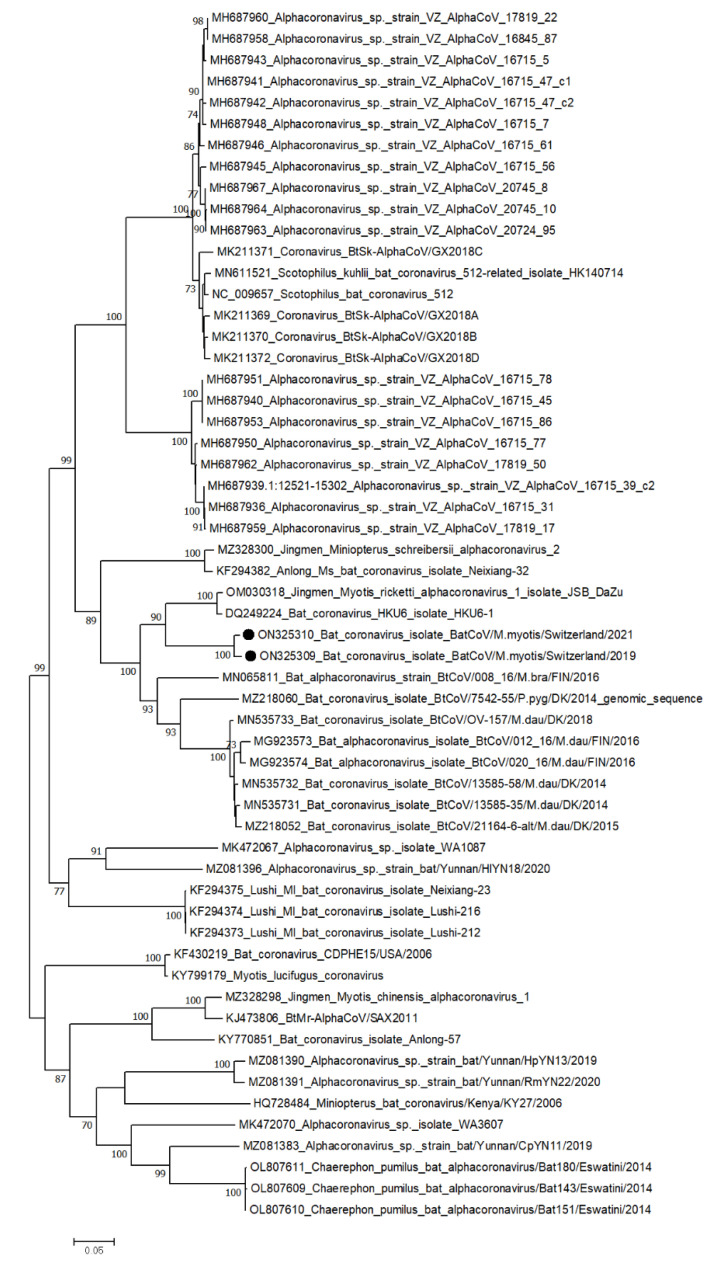
Phylogenetic tree based on nucleotide sequence identity of the NS12 protein (which includes *RdRp*) of selected alpha-coronaviruses. Sequences found in this study are marked by black dots. Phylogenetic analysis was performed using the Maximum likelihood algorithm based on the Tamura–Nei model with the 1000 replications bootstrap method. Only values ≥ 70% are displayed. Evolutionary analyses were conducted in MEGA X.

In the LU colony, sequences aligned to alpha-CoVs (MN535733 and MN535734) were found at all three time points and were chosen for further analysis. According to phylogenetic analysis based on the NS12 gene, these virus genomes clustered together in the phylogenetic tree and showed similarity (approx. 83%) to an alpha-CoV genome from China (OM030318), which is a member of the genus *alpha-coronavirus*, subgenus unclassified alpha-coronavirus (Figure 6). In the *ORF1ab*, 219 substitutions were detected, of which 35 were non-synonymous and 20 were novel (Table 2). In the gene encoding the spike protein, 99 substitutions were identified, of which 24 were non-synonymous and 23 were novel. In *ORF3,* 12 nucleotide substitutions were detected, of which 7 were non-synonymous and 2 were novel. In the gene encoding the envelope protein, only one substitution was detected, which was synonymous and not novel. Finally, the two substitutions in the gene encoding the nucleocapsid protein were both non-synonymous and novel (Table 2). Contigs from the de novo analysis were uploaded to GenBank under accession numbers ON325309 (2019; 27’932 nt, full genome) and ON325310 (2021; 27’259 nt, almost complete).

**Figure 7:**
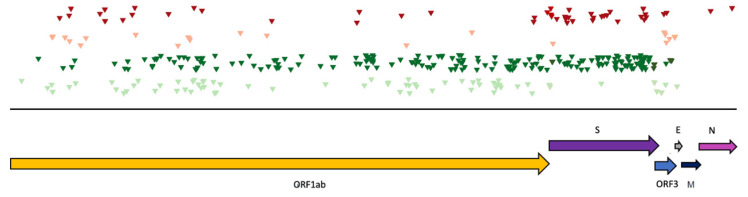
Overview of SNVs distributed over the genome of the alpha-CoV from the LU colony. Arrows show the position of the ORFs. Each triangle indicates an SNV; light green, synonymous and novel; dark green, synonymous; light red, non-synonymous and novel; dark red, non-synonymous.

In comparison to the MERS-related CoV from the AG colony, the substitutions were more evenly distributed over the genome in alpha-CoV from the LU colony and were more frequently non-synonymous (Figure 4 and Figure 7). However, in alpha-CoV, the nucleocapsid protein was less covered in the sequence from 2020. Of the 230 substitutions detected in the MERS-related CoV, 26 (11.3%) were non-synonymous, while of the 333 substitutions in the alpha-CoV genome, 68 (20.4%) were non-synonymous. The number of novel substitutions detected was comparable in alpha-CoV (47, 14.1%) and MERS-related CoV (25, 10.9%) (Table 2, Figure 4 and Figure 7).

## 4. Discussion

NGS and metagenomic analysis of ground stool samples of three different bat colonies of the species *Rhinolophus hipposideros, Vespertilio murinus*, and *Myotis myotis* sampled over three years identified genome sequences of 22 different virus families infecting vertebrates or invertebrates. A comparable number of virus families infecting vertebrates has been found in similar studies from the United States, South Africa, and French Guiana [35,36,58]. Viruses of vertebrates detected in our study, i.e., *Coronaviridae, Adenoviridae, Circoviridae*, and *Parvoviridae*, have been repeatedly detected in studies from Croatia, the United States, and China [12,34,36,40,59]. However, as opposed to these other reports, genomes of viruses that belong to the *Rhabdoviridae, Retroviridae,* and *Poxviridae* were not detected in our study. *Circoviridae* and *Genomoviridae* were the only viruses of vertebrates detected in all colonies and at all time points. Virus genomes from these two families were also the most abundant in a recent study from Argentina [37]. Nonetheless, direct comparison of viral diversity between various studies is challenging, since many factors, i.e., sample size, type, preparation, in silico analysis, bat species, location, and health of the bats, may affect virus composition.

Insectivorous bats hunt for prey in areas close to their roosting place. The presence and abundance of particular types of insects changes across seasons and from year to year. Indeed, no single virus species has been detected at two time points, although the diversity of viruses from invertebrates detected in each colony in our study was vast and several members of *Iflaviridae* and *Parvoviridae* were present in all years and all colonies (Figure 1). Viruses belonging to the family *Iflaviridae* can infect members of the orders *Lepidoptera, Hymenoptera, Hemiptera*, and *Diptera* [60,61,62,63,64,65,66]. Members of *Parvoviridae*, subfamily *Densovirinae*, infect invertebrates of six orders, including *Blattodea*, *Diptera*, *Hymenoptera*, *Hemiptera*, *Lepidoptera*, and *Orthoptera* [67], which are the main source of food for the bat species analyzed here [27]. In the GR colony, a high abundance of members of the subfamily *Densovirinae* was detected, similar to a previous study from China that also analyzed fecal samples [68].

The composition of virus genomes was similar in the three colonies, although each colony consisted of a different bat species. The ratio of virus families infecting vertebrates and invertebrates was 10 to 8 for AG and LU, and 9 to 9 for GR (Figure 1). Of note, some virus families consist of members that infect vertebrates (e.g., subfamily *Parvovirinae* within *Parvoviridae*) and other members that infect invertebrates (e.g., *Densovirinae* within *Parvoviridae*). Some virus families were detected in all colonies at least once. Among viruses infecting vertebrates, these included *Circoviridae*, *Genomoviridae*, *Parvoviridae*, *Adenoviridae*, *Picornaviridae*, and unclassified viruses. Among viruses infecting invertebrates, these included the *Iflaviridae*, *Dicistroviridae*, *Parvoviridae*, *Polycipiviridae*, and *Genomoviridae*. Viruses belonging to *Circoviridae, Adenoviridae*, and *Parvoviridae* are frequently detected in bats all over the world regardless of the species [8,9,36,58,69,70]. In our study, only *Genomoviridae*, *Circoviridae*, *Iflaviridae,* and *Parvoviridae* were detected in all colonies and at all time points. *Adenoviridae*, *Parvoviridae*, *Dicistroviridae*, and *Genomoviridae* were present at three time points in one colony (LU) and at two time points in the AG (likely because of the low read number obtained from that colony in 2020) and GR colonies (only two sampling time points available). Some virus families, e.g., *Polyomaviridae* and *Nairoviridae*, were detected just once or in two non-consecutive years. *Nairoviridae*, especially the genus Orthonairovirus, are known to use arthropods as hosts, and to be transmitted from there to mammals, birds, and bats [71]. Members of this family were found in the *Vespertilio murinus* bats of the AG colony in 2020 as well as in several other bat species in different countries, such as *Eptesicus nilsonii* in Germany and *Pipistrellus pipistrellus* and *Myotis mystacinus* in France [31,72]. Similarly, members of the family *Polyomaviridae* are well known to infect bats of multiple species and countries [33,73,74,75] and were also detected at all time points in the AG and LU colonies in our study. In the GR colony, polyomaviruses were not detected, although these viruses have been detected previously in bats of the genus *Rhinolophus* [76]. The differences found between the colonies may be due to the differences concerning the bat species, the environment, the hunting territory, and/or food availability.

Migratory behavior may increase contact with animals and pathogens and consequently increase the number of viruses. However, in our analysis of sedentary (*Rhinolophus hipposideros* and *Myotis myotis*) and migrating species (*Vespertilio murinus*), no gross differences in relative numbers of viral sequences were observed. It has previously been shown that the virome of migrating species is not remarkably abundant, and that migration can indeed lower infection risk because animals leave contaminated habitats, and infected and sick animals may remain behind or may not survive migration [39,77].

*Coronaviridae* members were consistently detected in the colonies of AG and LU over the three years. Both alpha- and beta-CoVs were found in previous studies performed on bat samples from many locations in Europe [78,79,80,81,82,83,84]. MERS-related-CoV was detected at all three time points, with the highest number of reads in 2020; the number of reads slightly decreased in 2021. So far, no human-pathogenic beta-CoVs have been isolated from bats in Europe. However, the viruses previously identified share 76–83% similarity with SARS-CoV, MERS-CoV, and SARS-CoV-2 [43,83,85,86]. The MERS-related-CoV sequence consistently found in all three years in the AG colony in our study showed the highest similarity (approximately 87%) to a MERS-related-CoV genome from China (MG021452). The AG colony is not accessible to the public, and contact with intermediate hosts in this region, and therefore a spillover event, is rather unlikely. It has been demonstrated that bats are the original reservoir host of MERS-CoV, and that dromedaries or camels can serve as intermediate hosts [18,87,88,89]. MERS-CoV uses the receptor human-dipeptidyl-peptidase-4 (hDPP4) for cell entry; however, most of the circulating MERS-related-CoV strains found in bats cannot bind hDPP4 properly and therefore likely use another receptor for cell entry [90,91]. Most importantly, MERS-related CoVs are not known to infect humans [85,90]. Nevertheless, Lau et al. showed that *Tylonicteris*-bat-CoV-HKU4 (*Ty*-BatCoV-HKU4), a *Merbecovirus* isolated from bats in southern China, can use hDPP4 as well as dromedary-camel-DPP4 (dc-DPP4) and *Tylonicteris pachypus*-DPP4 (*Tp*-DPP4) for cell entry and can infect hDPP4-transgenic mice [92,93]. As MERS-CoV can use only hDPP4 and dc-DPP4 but not *Tp*-DPP4 for cell entry, this indicates a risk that *Ty*-BatCoV-HKU4 may overcome the species barrier and may have the potential for direct bat-to-human transmission [93].

RNA viruses are predominant in the emergence of new diseases due to high mutation rates, which lead to the appearance of new mutants with increased fitness, highly diverse populations, and finally, easier adaptation to new hosts and outbreak events [94,95]. During the SARS-CoV pandemic, the virus may have been transmitted from bats to civets (an amplification host) and finally to humans [16,96]. Therefore, tracing the evolution of RNA viruses, especially CoVs, is of utmost importance. Many CoV sequences have been found since the SARS pandemic in 2003, allowing investigation of virus development over time in different locations and hosts. Furthermore, multiple studies following the emergence of new variants in the human population and with focus on the mutations of viruses in vitro have been conducted [93,97,98,99,100,101,102,103,104]. However, in vitro studies are performed under sterile conditions without the influence of multiple viral and bacterial co-infections as is commonly reported in bat samples and that can be insignificant, detrimental, or even beneficial for some viruses. Furthermore, the exact number of replication cycles is known in laboratory settings, while in the natural environment it can be only estimated. This might be an advantage regarding the accuracy of results and the ability to compare between different studies because the circumstances can be neglected in the interpretation of the results. However, in vitro discoveries cannot always be applied to the natural situation. For example, Lau et al. showed evidence that MERS-CoV isolated from bats was able to directly infect cells via the hDPP4 receptor [93]. However, no such direct transmissions have been reported in the natural environment. Various factors in the natural environment can influence host and infection dynamics, i.e., stress, lack of food and habitat, high animal density, which enables the introduction of new variants in a population, or multiple coinfections within individuals, which can lead to recombination events. Furthermore, intrinsic factors such as sex, age, breeding, and general health status may play a role in virus infection and surveillance [2,24,25,26], factors which cannot be mimicked in in vitro conditions.

The most important aspect of our work is that we can investigate virus genome evolution, including that of alpha- and beta-CoVs, in the natural reservoir species and environment. The substitution rate of both alpha- and beta-CoVs detected in this study was within the range of previously described values from 2 × 10^−2^/nt/year to 1 × 10^−5^/nt/year [105,106,107]. The general substitution rate of MERS-related-CoV was slightly lower (3.8 × 10^−3^/nt/year) than that of alpha-CoV (5.9 × 10^−3^/nt/year). This difference may be due to the poorer coverage of MERS-related CoV in 2019. Moreover, the number of novel and non-synonymous substitutions in alpha-CoV was also higher than in MERS-related CoV. The absolute numbers of substitutions in both MERS-related- and alpha-CoV were highest in *ORF1ab*, followed by the genes encoding the spike protein and the nucleocapsid protein, and correlated well with the substitutions found in SARS-CoV-2 genomes of human origin [108]. The absolute numbers of substitutions correlated directly with the length of the genes. The relative numbers of substitutions in the alpha-CoV genome were the highest in the spike protein with 1.2 × 10^−2^/nt/year, followed by *ORF1ab* and the genes encoding the envelope and nucleocapsid proteins with 5.4 × 10^−3^/nt/year, 2.17 × 10^−3^/nt/year, and 7.9 × 10^−3^/nt/year, respectively. The spike protein is known as a mutational hotspot [109,110,111,112]. Due to poorer coverage of the spike protein sequences in MERS-related CoV, not many substitutions were detected by NGS. However, Sanger sequencing of RT-PCR products from the genome region encoding the spike protein amplified from samples collected in the three consecutive years showed 429 substitutions, of which 96 were non-synonymous.

These data cannot be directly compared to the NGS data, which provide high sequencing depth at each nucleotide, while the Sanger sequencing data represent “frozen” sequences assembled from five overlapping RT-PCR products that are likely derived from multiple virus genomes. Nevertheless, Sanger sequencing shows that the spike protein region of MERS-related CoV is also highly variable and contains regions with higher frequencies of substitutions. Most interestingly, comparable regions with mutational hotspots were also found in a previous study of the genomes of SARS-CoV-2 from humans [113], although the overall number of nt substitutions was much lower in that report compared to our data.

Many studies have been performed to better understand the mutation pattern of SARS-CoV-2 and the dynamics of CoV evolution in the human population worldwide [107,114,115]. Qualitatively, the mutation pattern of the virus genomes from bats analyzed in this study was surprisingly comparable to that of other CoVs from humans [105,106,108,109,111,112] despite the differences in physiology, lifestyle, and environmental factors of the two different species. Bats are known to harbor viruses without showing any clinical signs. Their immune system is believed to have adapted to their unique biological and physiological characteristics, such as flight, longevity, and formation of large colonies. Moreover, their immune system is able to adapt and cope with viral infections over a long time of co-evolution, as they phylogenetically belong to the oldest species of mammals. Indeed, bats can control viral replication more effectively than other mammals [8,116,117,118].

Bats live in large, closed colonies where introducing new viruses to the population is difficult. However, once a new virus has been introduced, it may infect many individuals, which can lead to an increased number of replications and variants. Nevertheless, strains from different locations of bat SARS-like-CoV have similarities of approx. 90%, including strains identified in Russia and Bulgaria [80,83]. Humans, on the other hand, have much more open social structures compared to bats. Travel and contact with many random individuals may lead to effective spreading of virus and rising numbers of variants. While in human studies, mostly a single CoV variant is analyzed since samples are collected from individuals, in bat colonies, we can anticipate that several circulating variants are sequenced from the collection of fecal mixture from many bats.

In the DNA viruses found in our study, the number of substitutions was, as expected, relatively small, and the majority were synonymous, with one exception. Of the 12 nucleotide substitutions found in the *rep* genes of AAV2, 10 were non-synonymous. It is well known that parvoviruses such as parvovirus B19 in humans mutate at a much higher frequency than other DNA viruses [119,120,121,122]. This appears to also hold true for parvoviruses in bats. 

Circovirus genomes were detected in all three colonies. The number of substitutions in the *cap* ORF of GR and AG was small and all were synonymous. However, of the substitutions found in the *rep* sequences analyzed, some were non-synonymous. The number of substitutions per location and year was higher in the circoviruses in our study, with a rate of 6.02 × 10^−4^/nt/year to 7.9 × 10^−3^/nt/year, compared to previously reported values in porcine circovirus 3 (PCV3) of 2.35 × 10^−5^/nt/year to 7.32 × 10^−4^/nt/year [123,124,125], but comparable to that of psittacine beak and feather disease virus (PBFDV) (3.41 × 10^−3^/nt/year, [126]). It has been suggested that circoviruses may tolerate higher substitution rates than other DNA viruses due to their small genomes and low probability of accumulating deletions [127,128]. The vast diversity among PBFDV genomes supports the theory that viral evolutionary rates are not just dependent on the activity of high-fidelity polymerase, but also on genome architecture. Specifically, the loop-like DNA structures found in PBFDV may provide important recombination sites [105,126].

The collection of ground stool samples is an easy, noninvasive tool to surveil endemic bat populations. The gathered metagenomics data expand our knowledge of the variability of virus composition over time and the evolution of specific virus genomes in a natural reservoir species. This approach, nevertheless, has some limitations. First, the outcome and validity of the results can vary depending on the coverage and sequencing depth. For example, only few reads from RVs were obtained in 2021, which did not allow us to draw any conclusions on virus evolution. Similarly, the coverage and sequencing depth specifically from the genome region encoding the spike protein of MERS-related CoV from the AG colony was highly variable between the three consecutive years, a problem that was only partially overcome by targeted Sanger sequencing. Second, the colonies were re-sampled after a long interval; thus, new strains may have been introduced into the population.

In conclusion, this study gives insight into the complex diversity, abundance, and evolution of the virome and of the genomes of particular viruses in three selected bat colonies and provides a basis for further studies investigating how viruses can change over time in the natural reservoir host and environment.

## Figures and Tables

**Table 1:** SNVs detected in selected DNA viruses found in the bat colonies. Genes are shown with the length and number of synonymous (s)/non-synonymous (ns) nucleotide (nt) substitutions.

Colony	Virus	Gene	Length	nt-Substitutions s/ns
AG	Bat circovirus	Rep	920nt	2/0
		Cap	845nt	5/0
	Bat-associated circovirus	Rep	920nt	10/3
		Cap	779nt	14/0
	Pipistrellus pipistrellus polyomavirus	LTAg	2’512nt	1/0
GR	AAV2	Rep68	1’609nt	0/2
		Rep78	1’865nt	0/2
		Rep20	937nt	0/2
		Rep52	1’193nt	0/2
	Starling circovirus	Cap	830nt	1/0
LU	Cyclovirus	Rep	830nt	0/6
	Circovirus	Rep	830nt	3/3
	Dependoparvovirus	Cap	494nt	3/1
	Bat AAV	Cap	2’175nt	2/2
	Murine-associated porcine bocavirus	NP1	599nt	1/0
		VP1	1’850nt	3/0

**Table 2:** SNVs detected in selected RNA viruses found in the bat colonies. Genes are shown with the length and the number of synonymous (s)/non-synonymous (ns) nucleotide (nt) and novel substitutions.

Colony	Virus	Gene	Length	nt-Substitutions s/ns	Novel
AG	MERS-related CoV	ORF1ab	21’322nt	172/14	14
		S	4’049nt	9/3	3
		ORF4a	284nt	0/1	1
		ORF4b	698nt	1/1	1
		ORF5	683nt	3/1	1
		E	248nt	2/0	0
		M	656nt	8/0	0
		N	1’301nt	8/2	2
		ORF8b	581nt	1/4	3
GR	Bat rotavirus	NSP3	942nt	1/0	0
		VP4	2’331nt	1/0	0
	Mammalian orthoreovirus	S1	1’417nt	0/1	1
		L3	3’901nt	1/0	0
LU	Alpha CoV	ORF1ab	20’230nt	184/35	20
		S	4’094nt	75/24	23
		ORF3	674nt	5/7	2
		E	230nt	1/0	0
		N	1’259nt	0/2	2

## Data Availability

The nucleotide sequences of coronaviruses detected in this study have been registered at GenBank under accession numbers ON325307–ON325310. All the raw sequencing data generated in this study were uploaded to the Sequence Read Archive (SRA) under accession number PRJNA835632. Supplementary Materials showing detailed results of this study are available online.

## Supplementary Materials

The following supporting information can be downloaded at: https://www.mdpi.com/article/10.3390/v14091911/s1, Figure S1: Heatmap of specific genomes of viruses from invertebrates detected in the AG colony; Figure S2: Heatmap of specific genomes of viruses from invertebrates detected in the GR colony; Figure S3: Heatmap of specific genomes of viruses from invertebrates detected in the LU colony; Table S1: Database of specific viruses used for the alignment; Table S2: The primer pairs used for sequencing of the spike protein locus of the MERS-related CoVs from the Aargau colony including the expected product length.
