# Supplementary material for: Virus Diversity, Abundance, and Evolution in Three Different Bat Colonies in Switzerland
Source: Viruses. 2022 Aug 29;14(9):1911. doi: 10.3390/v14091911 (PMC9505930; doi:10.3390/v14091911)
Supplement: Supplementary file 1 [file viruses-14-01911-s001.zip › Supplementary captation.pdf]

Figure S1. Heatmap of specific genomes of viruses from invertebrates detected in the AG colony aligned to a collection of reference sequences. The first column shows the number of reads in a logarithmic scale, the second RPM, and the third the percent genome coverage. The read numbers are represented in a gradient from black (low numbers) to light yellow (high numbers). For the percent genome coverage black indicates 0% and light-yellow

Figure S2. Heatmap of specific genomes of viruses from invertebrates detected in the GR colony aligned to a collection of reference sequences. The first column shows the number of reads in a logarithmic scale, the second RPM, and the third the percent genome coverage. The read numbers are represented in a gradient from black (low numbers) to light yellow (high numbers). For the percent genome coverage black indicates 0% and light-yellow 100

Figure S3. Heatmap of specific genomes of viruses from invertebrates detected in the LU colony aligned to a collection of reference sequences. The first column shows the number of reads in a logarithmic scale, the second RPM, and the third the percent genome coverage. The read numbers are represented in a gradient from black (low numbers) to light yellow (high numbers). For the percent genome coverage black indicates 0% and light-yellow 100%.

Table S1. Database of viruses specific to each colony used for the alignments. In white are marked vertebrate viruses and in grey invertebrate viruses.

Table S2. The primer pairs used for sequencing of the spike protein locus of the MERS-related CoVs from the Aargau colony including the expected product length.
