# Supplementary material for: Virus Diversity, Abundance, and Evolution in Three Different Bat Colonies in Switzerland
Source: Viruses. 2022 Aug 29;14(9):1911. doi: 10.3390/v14091911 (PMC9505930; doi:10.3390/v14091911)
Supplement: Supplementary file 1 [file viruses-14-01911-s001.zip › Table S1.pdf]

Colony	Accession number	Virus genome
	LC456717	Bat_associated_circovirus_10
	MT815982	Bat_associated_circovirus
	KX064237	Bat_bunyavirus_JTM
	MT734803	Bat_chaphamaparvovirus
	NC_035799	Bat_circovirus
	KM382272	Bat_circovirus_POA_2012_V
	JX065122	Bat_mastadenovirus
	LC385827	Bat_mastadenovirus
	NC_029902	Bat_mastadenovirus_WIV11
	KY302871	Circovirus_sp_
	MG596802	Middle_East_respiratory_syndrome_related_coronavirus__MERS_CoV_
	MG596803	Middle_East_respiratory_syndrome_related_coronavirus__MERS_CoV_
	MG021451	Middle_East_respiratory_syndrome_related_coronavirus__MERS_CoV_
	MG021452	Middle_East_respiratory_syndrome_related_coronavirus__MERS_CoV_
	KT732793	Pacific_flying_fox_faeces_associated_gemycircularvirus_2
	KC522090	Pipistrellus_bat_coronavirus_HKU5
	LC426677	Pipistrellus_pipistrellus_polyomavirus_1
	LC426707	Scotophilus_kuhlii_polyomavirus_2
	NC_030843	Washington_bat_picornavirus
	M37899	Aedes_aegypti_densovirus_1
	KJ817182	Aphid_lethal_paralysis_virus
	NC_040347	Bark_beetle_associated_genomovirus_1
	KY933259	Big_Sioux_River_virus
	NC_001411	Black_beetle_virus
AG	MK433236	Blackfly_genomovirus_4
	EF517505	Black_queen_cell_virus
	AF329080	Boolarra_virus
	AF375296	Casphalia_extranea_densovirus
	NC_025219	Cripavirus_NB_1_2011_HUN
	MH188015	Culex_Iflavi_like_virus_4
	NC_028489	Daeseongdong_virus_2
	NC_023842	Danaus_plexippus_plexippus_iteravirus
	AY594353	Dendrolimus_punctatus_virus
	MN328434	Diamondback_moth_iflavirus
	NC_025835	Dinocampus_coccinellae_paralysis_virus
	NC_005092	Ectropis_obliqua_picorna_like_virus
	EF690537	Flock_House_virus
	KX423453	Helicoverpa_armigera_stunt_virus
	NC_033140	Hubei_permutotetra_like_virus_4
	NC_033148	Hubei_picorna_like_virus_60
	KX884604	Hubei_tetragnatha_maxillosa_virus_9
	KX884676	Hubei_tetragnatha_maxillosa_virus_9
	KX883169	Hubei_tombus_like_virus_28
	MG963176	Iteravirus_sp_
	NC_015115	Mosquito_densovirus_BR_07
	MH477289	Myrmica_scabrinodis_virus_2
	KM598406	Odonata_associated_circular_virus_20
	NC_027917	Opsiphanes_invirae_iflavirus_1

	NC_003113	Perina_nuda_virus
	MN535735	Rhopalosiphum_padi_virus__bat_metagenome_
	KY465674	Sacbrood_virus
	NC_033173	Sanxia_water_strider_virus_9
	NC_018399	Sibine_fusca_densovirus
	MK733235	Sitobion_miscanthi_virus_1
	MK543949	Tetranychus_urticae_associated_ambidensovirus
	MF173065	Tick_associated_genomovirus_1
	NC_040326	Tubeweb_spider_associated_circular_virus_1
	NC_037062	Varroa_mite_associated_genomovirus_1
	NC_040584	Yongsan_picorna_like_virus_3
GR	GU226971	Bat_adeno_associated_virus_YNM__BtAAV_
	NC_038389	Bat_associated_circovirus_7
	NC_038390	Bat_associated_circovirus_8
	MT815981	Bat_associated_circovirus
	NC_038400	Bat_associated_cyclovirus_11
	NC_038395	Bat_associated_cyclovirus_4
	KX064237	Bat_bunyavirus_JTM
	MG600411	Bat_hepadnavirus
	KY962687	Bat_Hepatitis_B_virus
	NC_029902	Bat_mastadenovirus_WIV11
	NC_029898	Bat_mastadenovirus_WIV9
	MN433617	Bat_rotavirus
	MN433618	Bat_rotavirus
	MN433619	Bat_rotavirus
	MN433620	Bat_rotavirus
	MN433621	Bat_rotavirus
	MN433622	Bat_rotavirus
	MN433623	Bat_rotavirus
	MN433624	Bat_rotavirus
	MN433625	Bat_rotavirus
	MN433626	Bat_rotavirus
	MN433627	Bat_rotavirus
	KY302864	Circovirus_sp_
	KY302872	Circovirus_sp_
	JQ762360	Fruit_bat_parvovirus
	KU597751	Human_rotavirus_A
	KM043060	lesser_horseshoe_bat_adenovirus_1
	KX263309	Mammalian_orthoreovirus_1
	KX263308	Mammalian_orthoreovirus_1
	KX263307	Mammalian_orthoreovirus_1
	KX263311	Mammalian_orthoreovirus_1
	KX263310	Mammalian_orthoreovirus_1
	KX263312	Mammalian_orthoreovirus_1
	KX263314	Mammalian_orthoreovirus_1
	KX263316	Mammalian_orthoreovirus_1
	KX263315	Mammalian_orthoreovirus_1
	KU194671	Mammalian_orthoreovirus_2
	MW582622	Mammalian_orthoreovirus

	MW582623	Mammalian_orthoreovirus
	MW582624	Mammalian_orthoreovirus
	MW582625	Mammalian_orthoreovirus
	MW582626	Mammalian_orthoreovirus
	MW582627	Mammalian_orthoreovirus
	MW582629	Mammalian_orthoreovirus
	MW582630	Mammalian_orthoreovirus
	MW582631	Mammalian_orthoreovirus
	KM087105	Mammalian_orthoreovirus
	KM087106	Mammalian_orthoreovirus
	KM087107	Mammalian_orthoreovirus
	KM087108	Mammalian_orthoreovirus
	KM087109	Mammalian_orthoreovirus
	KM087110	Mammalian_orthoreovirus
	KM087112	Mammalian_orthoreovirus
	KM087113	Mammalian_orthoreovirus
	KM087114	Mammalian_orthoreovirus
	MG457078	Mammalian_orthoreovirus
	MG457079	Mammalian_orthoreovirus
	MG457080	Mammalian_orthoreovirus
	MG457081	Mammalian_orthoreovirus
	MG457082	Mammalian_orthoreovirus
	MG457083	Mammalian_orthoreovirus
	MG457084	Mammalian_orthoreovirus
	MG457085	Mammalian_orthoreovirus
	MG457086	Mammalian_orthoreovirus
	MG457087	Mammalian_orthoreovirus
	MG457098	Mammalian_orthoreovirus
	MG457100	Mammalian_orthoreovirus
	MG457101	Mammalian_orthoreovirus
	MG457102	Mammalian_orthoreovirus
	MG457103	Mammalian_orthoreovirus
	MG457105	Mammalian_orthoreovirus
	MG457106	Mammalian_orthoreovirus
	MG457107	Mammalian_orthoreovirus
	MG457108	Mammalian_orthoreovirus
	MG457110	Mammalian_orthoreovirus
	MG457111	Mammalian_orthoreovirus
	MG457112	Mammalian_orthoreovirus
	MG457113	Mammalian_orthoreovirus
	MG457115	Mammalian_orthoreovirus
	MG457116	Mammalian_orthoreovirus
	MG457118	Mammalian_orthoreovirus
	MG457119	Mammalian_orthoreovirus
	MG457120	Mammalian_orthoreovirus
	MG457121	Mammalian_orthoreovirus
	MG457122	Mammalian_orthoreovirus
	MG457123	Mammalian_orthoreovirus
	MG457124	Mammalian_orthoreovirus

	MG457125	Mammalian_orthoreovirus
	MG457126	Mammalian_orthoreovirus
	MG457127	Mammalian_orthoreovirus
	KT444524	Mammalian_orthoreovirus
	KT444522	Mammalian_orthoreovirus
	KT444526	Mammalian_orthoreovirus
	KT444525	Mammalian_orthoreovirus
	KT444527	Mammalian_orthoreovirus
	KT444531	Mammalian_orthoreovirus
	KT444530	Mammalian_orthoreovirus
	KT444581	Mammalian_orthoreovirus
	KT444572	Mammalian_orthoreovirus
	KT444574	Mammalian_orthoreovirus
	KT444575	Mammalian_orthoreovirus
	KT444576	Mammalian_orthoreovirus
	KT444577	Mammalian_orthoreovirus
	KT444579	Mammalian_orthoreovirus
	KT444580	Mammalian_orthoreovirus
	KT444534	Mammalian_orthoreovirus
	KT444532	Mammalian_orthoreovirus
	KT444536	Mammalian_orthoreovirus
	KT444535	Mammalian_orthoreovirus
	KT444537	Mammalian_orthoreovirus
	KT444541	Mammalian_orthoreovirus
	KT444540	Mammalian_orthoreovirus
	KT444544	Mammalian_orthoreovirus
	KT444542	Mammalian_orthoreovirus
	KT444546	Mammalian_orthoreovirus
	KT444545	Mammalian_orthoreovirus
	KT444547	Mammalian_orthoreovirus
	KT444551	Mammalian_orthoreovirus
	KT444550	Mammalian_orthoreovirus
	KT444554	Mammalian_orthoreovirus
	KT444552	Mammalian_orthoreovirus
	KT444556	Mammalian_orthoreovirus
	KT444555	Mammalian_orthoreovirus
	KT444557	Mammalian_orthoreovirus
	KT444561	Mammalian_orthoreovirus
	KT444560	Mammalian_orthoreovirus
	KT444564	Mammalian_orthoreovirus
	KT444563	Mammalian_orthoreovirus
	KT444562	Mammalian_orthoreovirus
	KT444566	Mammalian_orthoreovirus
	KT444565	Mammalian_orthoreovirus
	KT444567	Mammalian_orthoreovirus
	KT444569	Mammalian_orthoreovirus
	KT444571	Mammalian_orthoreovirus
	KT444570	Mammalian_orthoreovirus
	JQ412755	Mammalian_Orthoreovirus_strain_T3_Bat_Germany_342_08

	JQ412756	Mammalian_Orthoreovirus_strain_T3_Bat_Germany_342_08
	JQ412758	Mammalian_Orthoreovirus_strain_T3_Bat_Germany_342_08
	JQ412759	Mammalian_Orthoreovirus_strain_T3_Bat_Germany_342_08
	NC_038485	Pacific_flying_fox_faeces_associated_gemycircularvirus_2
	NC_038493	Pteropus_associated_gemycircularvirus_10
	NC_038488	Pteropus_associated_gemycircularvirus_5
	KC960619	Rotavirus_A
	MN539290	Rotavirus_A
	MT796864	Rotavirus_A
	LC576609	Rotavirus_A
	KC178799	Rotavirus_A
	HQ846846	Rotavirus_A
	MT226638	Rotavirus_A
	MT226640	Rotavirus_A
	KF690125	Rotavirus_A
	KU243602	Rotavirus_G3
	NC_030843	Washington_bat_picornavirus
	AY095351	Aedes_albopictus_C6_36_cell_densovirus
	JX045858	Aphid_lethal_paralysis_virus
	MK704471	Aphid_lethal_paralysis_virus
	MG571099	Bark_beetle_associated_genomovirus_3
	KX883903	Beihai_picorna_like_virus_76
	MK433234	Blackfly_genomovirus_2
	MK433238	Blackfly_genomovirus_6
	KP119603	Black_queen_cell_virus
	NC_005041	Blattella_germanica_densovirus_1
	JQ320376	Blattella_germanica_densovirus_like_virus
	KP714090	Bloomfield_virus
	KP714095	Bloomfield_virus
	MH614292	Boghill_Burn_virus
	MF953177	Brandeis_virus
	NC_004288	Casphalia_extranea_densovirus
	NC_025219	Cripavirus_NB_1_2011_HUN
	MH188043	Culex_densovirus
	MH188004	Culex_dicistrovirus_1
	NC_040574	Culex_Iflavi_like_virus_4
	MH188026	Culex_mosquito_virus_1
	MF176305	Culex_negev_like_virus_1
	NC_012685	Culex_pipiens_densovirus
	JQ659255	Culex_Y_virus
	KU095842	Daeseongdong_virus_2
	NC_023842	Danaus_plexippus_plexippus_iteravirus
	JN857348	Densovirus_SC3908
	NC_023871	Dragonfly_associated_circular_virus_2
	GQ342962	Drosophila_melanogaster_birnavirus_SW_2009a
	KU754527	Eridge_virus
	KU754528	Eridge_virus
	NC_016518	Espirito_Santo_virus
	NC_016517	Espirito_Santo_virus

	DNVCAPSID	Galleria_mellonella_densovirus
	NC_029052	Goose_dicistrovirus
	MH545499	Grasshopper_associated_circular_virus_1
	NC_024016	Heliconius_erato_iflavirus
	MF176264	Hubei_chryso_like_virus_1
	KX882764	Hubei_mosquito_virus_2
	NC_033157	Hubei_picorna_like_virus_58
	KC883978	Junonia_coenia_densovirus
	NC_007619	Kelp_fly_virus
	NC_015115	Mosquito_densovirus_BR_07
	JX403941	Mosquitoe_x_virus
	MH477289	Myrmica_scabrinodis_virus_2
	KM598406	Odonata_associated_circular_virus_20
	NC_038482	Odonata_associated_gemycircularvirus_1
	NC_027917	Opsiphanes_invirae_iflavirus_1
	NC_011588	Oryctes_rhinoceros_nudivirus
	NC_031450	Parus_major_densovirus
	NC_040344	Pimoid_spider_associated_circular_virus_2
	NC_034217	Pityohyphantes_rubrofasciatus_iflavirus
	JX645046	Pseudoplusia_includens_densovirus
	NC_033173	Sanxia_water_strider_virus_9
	NC_040338	Sierra_dome_spider_associated_circular_virus_1
	MK733235	Sitobion_miscanthi_virus_1
	NC_022748	Solenopsis_invicta_densovirus
	MH545503	Spider_associated_circular_virus_1
	NC_040368	Spinybacked_orbweaver_circular_virus_1
	NC_016405	Spodoptera_exigua_iflavirus_1
	MH545502	Termite_associated_circular_virus_2
	NC_033747	Thrips_associated_genomovirus_4
	KY457233	Tomelloso_virus
	NC_040326	Tubeweb_spider_associated_circular_virus_1
	KX884091	Wuhan_fly_virus_5
LU	MH170363	Artibeus_lituratus_parvovirus
	KX907135	Bastrovirus_like_virus_VietNam_Bat_17819_21
	MH167452	Bat_adeno_associated_virus
	GU226971	Bat_adeno_associated_virus_YNM__BtAAV_
	JN252129	Bat_adenovirus_2
	NC_016895	Bat_adenovirus_TJM__BtAdV_
	MG923574	Bat_alphacoronavirus
	MN065811	Bat_alphacoronavirus
	MT815980	Bat_associated_circovirus
	MN832787	Bat_astrovirus__bat_metagenome_
	MT734803	Bat_chaphamaparvovirus
	MN535734	Bat_coronavirus__bat_metagenome_
	MN535733	Bat_coronavirus__bat_metagenome_
	KY770850	Bat_coronavirus
	NC_038316	Bat_hepatovirus_BUO2BF86Colafr2010
	LC385828	Bat_mastadenovirus_A
	MF540585	Bat_mastadenovirus

KJ641665	Bat_parvovirus
MZ218061	Bat_picornavirus__bat_metagenome_
MN433617	Bat_rotavirus
MN433618	Bat_rotavirus
MN433619	Bat_rotavirus
MN433620	Bat_rotavirus
MN433621	Bat_rotavirus
MN433622	Bat_rotavirus
MN433623	Bat_rotavirus
MN433624	Bat_rotavirus
MN433625	Bat_rotavirus
MN433626	Bat_rotavirus
MN433627	Bat_rotavirus
KJ473806	BtMr_AlphaCoV_SAX2011
KY302869	Circovirus_sp_
KY302871	Circovirus_sp_
MF579866	Dependoparvovirus_sp_
KX907333	Desmodus_rotundus_parvovirus
KM043106	greater_mouse_eared_bat_adenovirus_1
JF304919	Human_rotavirus_A
NC_029797	Megabat_bufavirus_1
LC426674	Myotis_horsfieldii_polyomavirus_1
NC_039045	Myotis_myotis_bocavirus_1
KC154060	Myotis_ricketti_parvovirus
KF294381	Neixiang_Md_bat_coronavirus
KT732793	Pacific_flying_fox_faeces_associated_gemycircularvirus_2
JF755426	Peromyscus_adeno_associated_virus_M_6_USA_2010
NC_038493	Pteropus_associated_gemycircularvirus_10
NC_038489	Pteropus_associated_gemycircularvirus_6
LC426696	Rhinolophus_sinicus_polyomavirus_1
KX420939	Rotavirus_A
KC960620	Rotavirus_A
KC960622	Rotavirus_A
KX814956	Rotavirus_A
MN528120	Rotavirus_A
MN528105	Rotavirus_A
MN567261	Rotavirus_A
NC_030843	Washington_bat_picornavirus
MN565031	Acute_bee_paralysis_virus
MN099038	Ambidensovirus_Croatia_17_S17
MK704471	Aphid_lethal_paralysis_virus
KJ817182	Aphid_lethal_paralysis_virus
MF458893	Aphid_lethal_paralysis_virus
MN510869	Aphid_lethal_paralysis_virus
MT096523	Aphid_lethal_paralysis_virus__insect_metagenome_
NC_040347	Bark_beetle_associated_genomovirus_1
MG571097	Bark_beetle_associated_genomovirus_6
KX644942	Bat_cripavirus
NC_035139	Bemisia_associated_genomovirus_NfO

	MK433234	Blackfly_genomovirus_2
	MT416539	Black_queen_cell_virus
	NC_005041	Blattella_germanica_densovirus_1
	AF375296	Casphalia_extranea_densovirus
	NC_025219	Cripavirus_NB_1_2011_HUN
	MH188043	Culex_densovirus
	MH545507	Cybaeus_spider_associated_circular_virus_2
	NC_023842	Danaus_plexippus_plexippus_iteravirus
	MN565037	Deformed_wing_virus__DWV_
	NC_001899	Diatraea_saccharalis_densovirus
	MN918742	Dicistroviridae_sp___bird_metagenome_
	MN905969	Dicistroviridae_sp___bird_metagenome_
	MN917664	Dicistroviridae_sp___bird_metagenome_
	MN905986	Dicistroviridae_sp___bird_metagenome_
	NC_023871	Dragonfly_associated_circular_virus_2
	KU754505	Empeyrat_virus
	NC_023021	Formica_exsecta_virus_1
	NC_040346	Giant_house_spider_associated_circular_virus_1
	NC_029052	Goose_dicistrovirus
	AY605055	Haemagogus_equinus_densovirus
	NC_040611	Hedgehog_dicipivirus
	JQ894784	Helicoverpa_armigera_densovirus
	MH310078	Hypsignathus_monstrosus_dicistrovirus
	NC_023615	Invertebrate_iridescent_virus_22
	NC_023613	Invertebrate_iridovirus_25
	KC690269	Israeli_acute_paralysis_virus__IAPV_
	KY465690	Israeli_acute_paralysis_virus__IAPV_
	MG963176	Iteravirus_sp_
	KC883978	Junonia_coenia_densovirus
	AY275710	Kashmir_bee_virus
	NC_035450	Lasius_neglectus_virus_1
	NC_035456	Lasius_niger_virus_1
	NC_035457	Myrmica_scabrinodis_virus_1
	MH477289	Myrmica_scabrinodis_virus_2
	NC_005341	Mythimna_loreyi_densovirus
	NC_038482	Odonata_associated_gemycircularvirus_1
	NC_034217	Pityohyphantes_rubrofasciatus_iflavirus
	MW826528	Polycipiviridae_sp_
	NC_034217	Pityohyphantes_rubrofasciatus_iflavirus
	MW826528	Polycipiviridae_sp_
	MG545287	Sacbrood_virus
	NC_018399	Sibine_fusca_densovirus
	NC_040338	Sierra_dome_spider_associated_circular_virus_1
	MH545504	Spider_associated_circular_virus_1
	MH545502	Termite_associated_circular_virus_2
	NC_033742	Thrips_associated_genomovirus_3
	MF173067	Tick_associated_genomovirus_3
	AY251269	Varroa_destructor_virus_1
	NC_037062	Varroa_mite_associated_genomovirus_1

	NC_015780	Wiseana_iridescent_virus
