# Supplementary material for: Virus Diversity, Abundance, and Evolution in Three Different Bat Colonies in Switzerland
Source: Viruses. 2022 Aug 29;14(9):1911. doi: 10.3390/v14091911 (PMC9505930; doi:10.3390/v14091911)
Supplement: Supplementary file 1 [file viruses-14-01911-s001.zip › Table S2.pdf]

| Year | Forward | Reverse | Product length |
|---|---|---|---|
| 2019 | 5'-GCTCTCAGCGTGTCTACTGATG-3' | 5'-ACCACTCAGCGTTTTCATCCTC-3' | 791nt |
| | 5'-TACGTCGTTTGGGAAACACC-3' | 5'-CAACATTGGTGTCATTTCGCAC-3' | 1166nt |
| | 5'-TCATCTCTGTTCAGTATGGCAC-3' | 5'-RCCAGGRTCAGCAATAGTAACC-3' | 977nt |
| | 5'-TGGTGCTAATCTTAGACARGATG-3' | 5'-AGAAGTAAARCCCGTYTGGWG-3' | 902nt |
| | 5'-AAATGGATTYTGCGGATCTGG-3' | 5'-TGGACATGAATCTTATGTGGCTC-3' | 726nt |
| 2020 | 5'-ACTCTCARYGTKTCTACTGATG-3' | 5'-GATGCTGCGTGGAATGACTG-3' | 919nt |
| | 5'-AGAAAAGGGGACTTGTATGGTGG-3' | 5'-ACATTGGTGTCATTTCGCACAG-3' | 944nt |
| | 5'-TCATCTCTGTTCAGTATGGCAC-3' | 5'-RCCAGGRTCAGCAATAGTAACC-3' | 977nt |
| | 5'-TGGTGCTAATCTTAGACARGATG-3' | 5'-AGAAGTAAARCCCGTYTGGWG-3' | 902nt |
| | 5'-AAATGGATTYTGCGGATCTGG-3' | 5'-TGGACATGAATCTTATGTGGCTC-3' | 726nt |
| 2021 | 5'-ACTCTCARYGTKTCTACTGATG-3' | 5'-GATGCTGCGTGGAATGACTG-3' | 919nt |
| | 5'-CCTCTTTGCATCTTTGCCTG-3' | 5'-AGWGCAGTTWTGRAAGACACC-3' | 1000nt |
| | 5'-GGCTGTTCGCAACGATACAAAG-3' | 5'-CCAGTCAAACCTGCATCAAGC-3' | 812nt |
| | 5'-TGGTGCTAATCTTAGACARGATG-3' | 5'-AGAAGTAAARCCCGTYTGGWG-3' | 902nt |
| | 5'-AAATGGATTYTGCGGATCTGG-3' | 5'-TGGACATGAATCTTATGTGGCTC-3' | 726nt |
