# Supplementary figures and images for: Virus Diversity, Abundance, and Evolution in Three Different Bat Colonies in Switzerland
Source: Viruses. 2022 Aug 29;14(9):1911. doi: 10.3390/v14091911 (PMC9505930; doi:10.3390/v14091911)

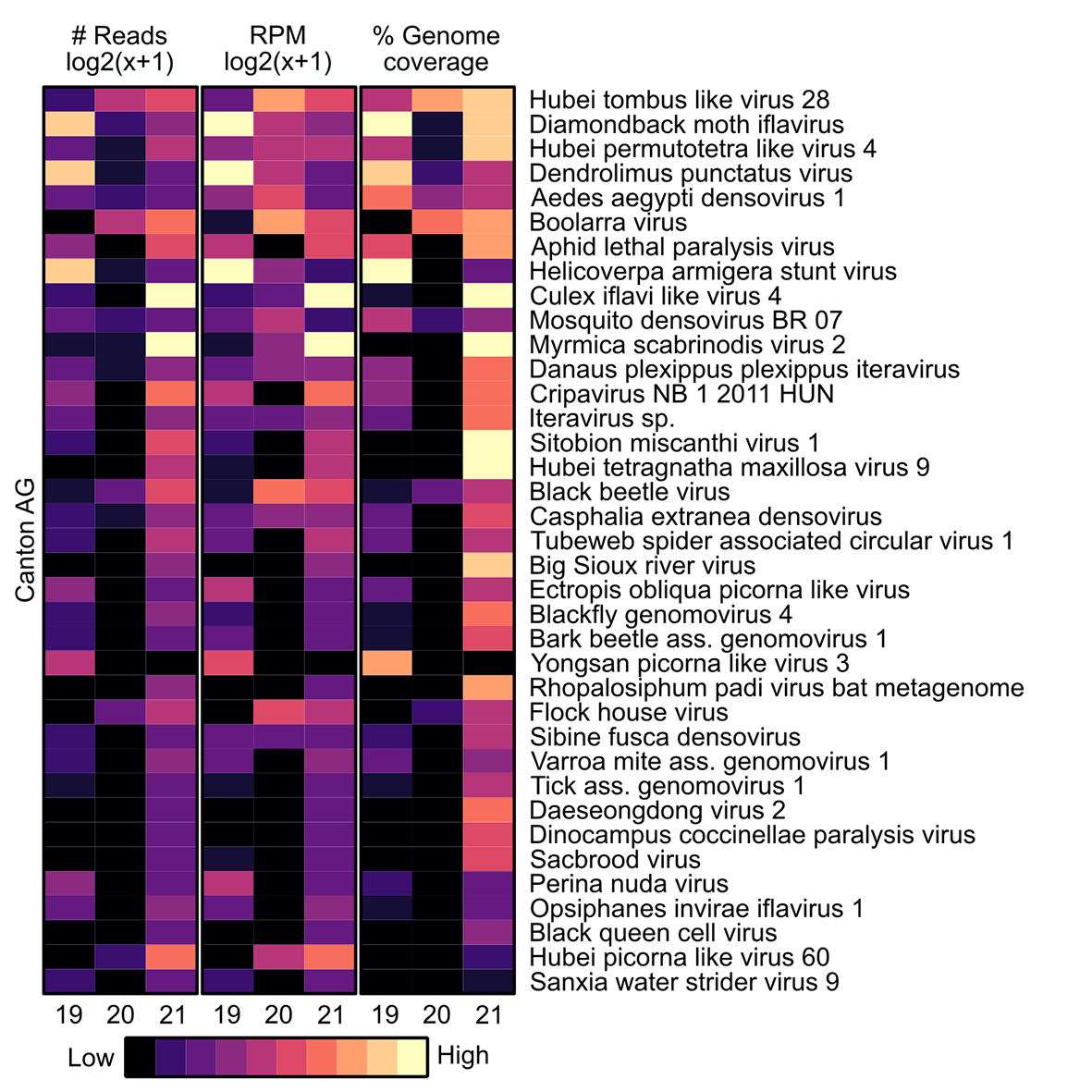

Supplement: Supplementary file 1 [file viruses-14-01911-s001.zip › Figure S1.tif]

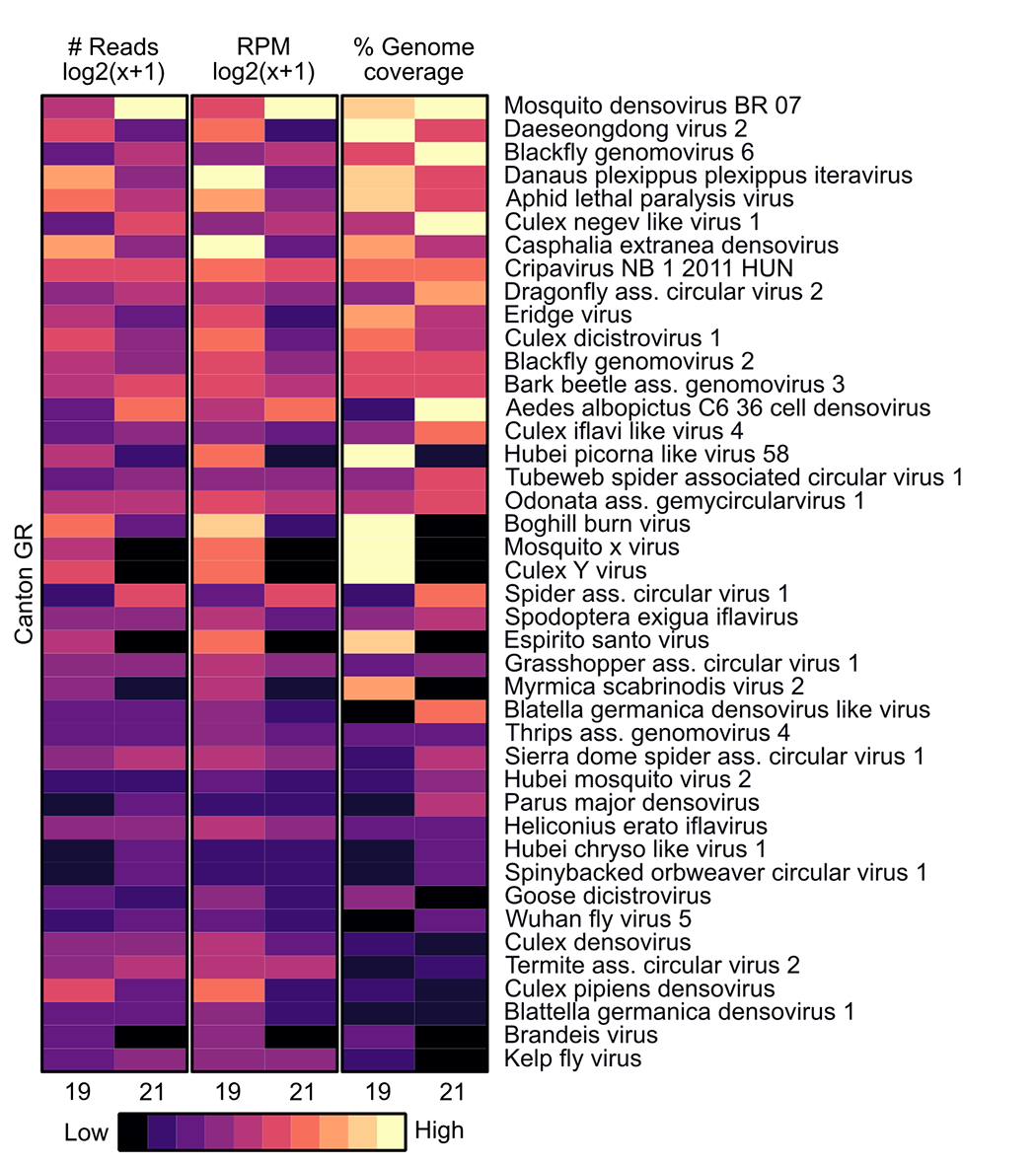

Supplement: Supplementary file 1 [file viruses-14-01911-s001.zip › Figure S2.tif]

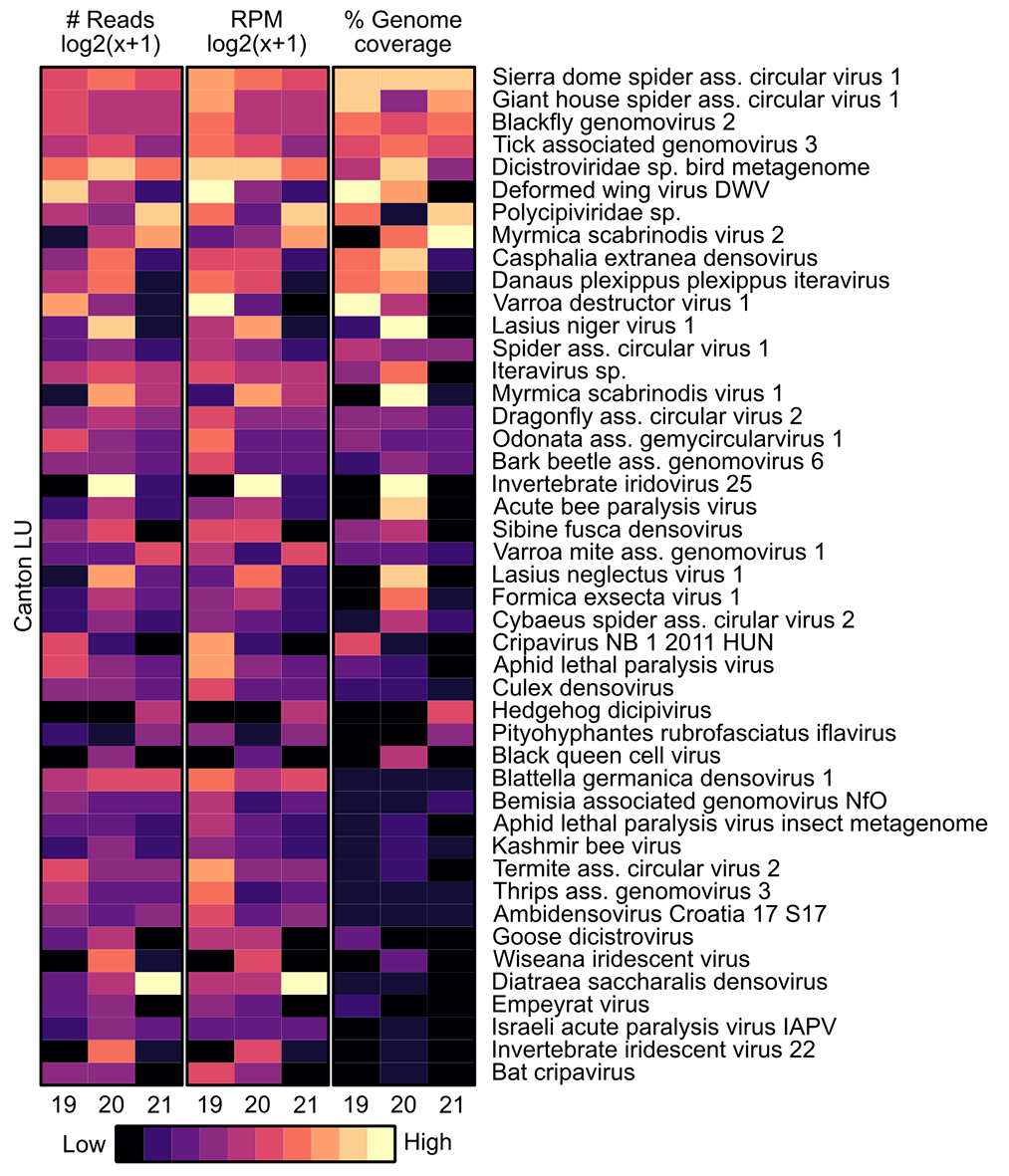

Supplement: Supplementary file 1 [file viruses-14-01911-s001.zip › Figure S3.tif]
